# Analysis of Gremlin 1 Levels Following Sleeve Gastrectomy

**DOI:** 10.7759/cureus.48738

**Published:** 2023-11-13

**Authors:** Khalid A Alregaiey, Assim A Alfadda, Naif S Alsaber, Abdulrahman M Bedaiwi, Faris R Almubarak, Abdullah F Bin Muammar, Rakan A Alfaifi, Mohammed N Alquwayfili, Rahma M Alyami, Muhammad Iqbal

**Affiliations:** 1 Physiology, King Saud University, Riyadh, SAU; 2 Internal Medicine, King Saud University, Riyadh, SAU

**Keywords:** insulin resistance, obesity, insulin sensitivity, sleeve gastrectomy, gremlin 1

## Abstract

Background: In the current study, we aimed to assess the levels of Gremlin 1, an adipokine with a rich repertoire of metabolic effects, in association with the glycemic and lipid parameters after sleeve gastrectomy.

Material and methods: This study was conducted on 31 males with obesity aged 25 to 50 years who underwent sleeve gastrectomy. Plasma Gremlin 1 levels were evaluated using enzyme-linked immunosorbent assay (ELISA) at baseline and 6-12 months after the operation, along with body mass index, insulin, glucose, and lipid profile.

Results: Plasma Gremlin 1 levels were elevated (148.19±17.43 vs. 193.29±19.82 ng/mL, p < 0.05) after sleeve gastrectomy. This was accompanied by a decrease in body mass index (from 51.47±1.71 to 39.23±1.56 kg/m^2^, p < 0.05). Insulin and Homeostatic Model Assessment for Insulin Resistance (HOMA-IR) also exhibited a significant decrease (19.69±1.81 vs. 8.98±1.09 mIU/L and 6.52±0.98 vs. 2.57±0.036 p < 0.05, respectively) in the postoperative period. Total cholesterol levels were significantly increased after surgery (4.29±0.16 to 5.10±0.16, p < 0.05). Pearson correlation analysis showed that Gremlin 1 was positively correlated with insulin before surgery, but there was no significant correlation after surgery.

Conclusion: The circulating Gremlin 1 levels were elevated postoperatively among our participants. The improvement in insulin sensitivity appears to be independent of the reported antagonistic effects of Gremlin 1.

## Introduction

Obesity has long been associated with insulin resistance, an ominous condition characterized by the relative insensitivity of a target cell or a whole organ to insulin [1]. Means to achieve weight loss have attracted considerable interest in determining the most effective methods for avoiding the potentially catastrophic effects of obesity. Bariatric surgery is now perceived as the most effective method for weight loss and remission of type 2 diabetes compared to non-surgical methods [2].

Of interest to this study was laparoscopic sleeve gastrectomy, an operation that involves the surgical resection of roughly two-thirds of the stomach, resulting in a restriction of gastric volume and an earlier sensation of fullness. In addition to the obvious effect of combating obesity, various studies have also helped to elucidate the role of bariatric surgery in alleviating the risk of other potentially grave health conditions, which are not necessarily reflective of the beneficial effects of weight loss, but of a profound change in metabolic markers potentiated by the yet undiscovered effects of bariatric surgery. These studies also reported remission of hypertension and hyperlipidemia and a reduced risk of developing atherosclerosis following bariatric surgery. Consequently, more questions have been raised about the mechanisms underlying these results [3-6].

Gremlin 1, is a glycoprotein consisting of 184 amino acids with a rich repertoire of biological effects, including renal organogenesis, limb morphogenesis, and improved regenerative potential of cardiac cells [7-8]. Gremlin 1 is also a known antagonist of bone morphogenic proteins, such as bone morphogenetic protein 4 (BMP4) and bone morphogenetic protein 7 (BMP7), a group of anti-inflammatory growth factors of the transforming growth factor-β superfamily [9]. It has been speculated that elevated levels of Gremlin 1 in adiposity tissue contribute to the inflammatory effects and apparent adipose tissue failure observed in these individuals.

Gremlin 1 binds to soluble BMP4 and BMP7 to inhibit their interaction with their complement receptors. BMP4 and BMP7 are known to be inducive of the browning process, which takes place in white adipose tissue (WAT), resulting in the so-called “beige” adipose tissue [10-11]. BMP4 was prominently shown to be secreted by expanded adipose tissue to drive the commitment of adipose mesenchymal cells into the WAT lineage, thus acting as an adipogenic regulator for lipid reservoirs [12]. Overall, this adipogenic role, coupled with the finding that such brown-like adipose tissue improves beta-cell function through the attenuation of insulin demands, has made BMPs attractive targets for the treatment of type 2 diabetes [13-15]. Elevated levels of Gremlin 1 in obesity may theoretically account for the decreased volume of beige and/or brown adipose tissue in obesity, and possibly a reduced glucose-buffering capacity of beige and/or brown adipose tissue, resulting in reduced insulin sensitivity. Reports of such a relationship have consistently illustrated this role, whereby Gremlin 1 was found to be the main antagonist of BMP4-driven beige adipose tissue activation, with the restoration of brown adipose tissue markers after silencing Gremlin 1, as well as antagonizing the anti-senescent, anti-steatotic, anti-inflammatory, and anti-fibrotic effects of BMP4 [16-17]. While Gremlin 1 is pro-senescent and antagonistic to BMP4 in hepatocytes [17], BMP4 was recently reported to reduce hepatic glucose levels through activation of the mTORC2 signaling pathway, implying an important role of BMP4 in regulating hepatic glucose metabolism [18]. Collectively, these results indicate that Gremlin 1 may contribute to increased hepatic glucose production by antagonizing BMP4 expression. Furthermore, we recently reported that higher plasma Gremlin 1 levels correlated positively with poor glycemic control and fat mass in females with type 2 diabetes [19].

Hedjazifar et al. have reported a decline in Gremlin 1 mRNA expression in subcutaneous and visceral adipose tissues, following a two-step bariatric surgery in 55 individuals with morbid obesity, along with evidence of insulin antagonism, which was effectively neutralized in vitro by the addition of anti-Gremlin 1 antibody [20]. These observations suggest an interesting contribution of Gremlin 1 in obesity Therefore, in our study, we aimed to investigate the changes in plasma Gremlin 1 levels and their association with glycemic parameters after sleeve gastrectomy.

## Materials and methods

Ethical statement

This study was conducted in accordance with the ethical principles of the Declaration of Helsinki. The research protocol was analyzed and approved (E-20-5425) by the Institutional Review Board and Ethics Committee of King Saud University College of Medicine. Written consent was obtained from all participants. No rewards or incentives were provided for participation in this study.

Study design and subjects

This study was conducted at the Department of Physiology and Obesity Research Center, College of Medicine, King Saud University. This study included 31 healthy male individuals aged 25-49 years who underwent sleeve gastrectomy. All patients enrolled in this study were routinely examined as part of the preoperative bariatric surgery evaluation by a nutritionist, physician, and psychologist before the surgical intervention. A follow-up period of 6-12 months postoperatively was chosen with respect to the date of the operation of each participant.

Analysis of Gremlin 1 by enzyme-linked immunosorbent assay (ELISA) and biochemical assessments

Blood was collected from patients one day before surgery and during the 6-12-month follow-up period. Plasma was separated and stored in aliquots at -80ºC. Other parameters, such as insulin, glucose, and lipid profiles, were retrieved from hospital files at the same time points. Plasma levels of Gremlin 1 in patients (31 pre- and post-sleeve gastrectomies) were analyzed using simple-step human ELISA kits (GREM1, Cat. #: abx151737) following the manufacturer’s instructions (Abbexa, Cambridge, UK). Briefly, plasma samples and assay standards were loaded into a 96-well plate pre-coated with antibodies and incubated at 37 °C for 90 min. Then, 100 μL of detection reagent A was added and incubated at 37°C for 1 h. The assay plate was washed three times with wash buffer, detection reagent B was added, and the incubation continued for 30 min at 37°C. After washing five times, 3,3′,5,5′-tetramethylbenzidine (TMB) substrate was added and incubated for 20 min at 37°C. The reaction was stopped by aliquoting 50 µL of stop solution into each well and the absorbance was read by a microplate reader (EL 800, BioTek Instruments, USA) at 450 nm.

Data analysis

Bivariate statistical results were analyzed using a paired t-test to compare data before and after sleeve gastrectomy. Numerical data are expressed as the mean and standard error of the mean, and categorical data are expressed as numbers and percentages. A two-tailed analysis was utilized for which a p-value of ≤ 0.05 was considered significant. Correlation analysis was used to compare the parameters. Numerical data were analyzed using SPSS Statistical Software 27.0 (IBM Corp., Armonk, NY).

## Results

Demographic and clinical assessment 

Body mass index (BMI) was significantly decreased after surgery, from 51.47±1.71 to 39.23±1.56 kg/m^2^, p < 0.05 (Table 1). Our findings also showed a significant reduction in the Homeostatic Model Assessment for Insulin resistance (HOMA-IR) index (from 6.52±0.98 to 2.57±0.036), following surgery (p < 0.05). Moreover, there was a significant decline in circulating insulin levels (19.69±1.81 vs. 8.98±1.09 mIU/L, p < 0.05) following surgery with an insignificant decrease in fasting plasma glucose in the follow-up period (p = 0.196). Serum triglyceride levels declined following bariatric surgery (from 1.63±0.22 to 1.06±0.77 mmol/L, p < 0.05). After the surgery, there was an insignificant increase in low-density lipoprotein (LDL) level compared to baseline levels (from 2.64±0.15 to 2.97±0.13 mmol/L, p=0.056); however, there was a significant increase in high-density lipoprotein (HDL) levels after surgery (0.89±0.04 to 1.55±0.06 mmol/L, p < 0.05). Likewise, there was an increase in plasma cholesterol levels following sleeve gastrectomy compared to baseline levels, from 4.29±0.16 to 5.10±0.16, p < 0.05 (Table 1). Plasma albumin, calcium, phosphorus, and vitamin D levels were significantly elevated after sleeve gastrectomy, whereas parathyroid hormone levels were not altered (Table 1).

**Table 1:** Pre-operative and post-operative clinical and plasma biochemical parameters Note: Values of p < 0.05 indicate statistical significance. BMI, body mass index; GLUC, glucose; TGL, triglycerides; CHOL, cholesterol; HDL, high-density lipoprotein; LDL, low-density lipoprotein; HOMA-IR, homeostatic model assessment for insulin resistance; Corr, calcium-corrected; PTH, Parathyroid hormone.

Parameters	Before surgery Mean ± SEM	After surgery Mean ± SEM	p-value
BMI (kg/m^2^)	51.47± 1.71	39.23± 1.56	0.001
GLUC (mmol/L)	7.02 ± 0.50	6.30 ± 0.19	0.196
TGL (mmol/L)	1.63 ± 0.22	1.06 ± 0.07	0.029
CHOL (mmol/L)	4.29 ± 0.16	5.10 ± 0.16	0.001
HDL (mmol/L)	0.89 ± 0.04	1.55 ± 0.06	0.001
LDL (mmol/L)	2.64 ± 0.15	2.97 ± 0.13	0.056
Insulin (mIU/L)	19.69 ± 1.81	8.98 ± 1.09	0.001
HOMA-IR index	6.52 ± 0.98	2.57 ± 0.36	0.001
Albumin (g/L)	37.15±0.81	43.04±0.80	0.001
Total calcium (mmol/L)	2.16±0.02	2.51±0.02	0.001
Corr calcium (mmol/L)	2.22±0.01	2.41±0.02	0.001
Phosphorus (mmol/L)	1.13±0.04	1.44±0.03	0.001
PTH (pmol/L)	7.39±0.50	6.30±0.50	0.060
Vitamin D (ng/mL)	8.77±0.86	19.87±1.79	0.001

Analysis of Gremlin 1 and its correlation with clinical parameters

**Figure 1:**
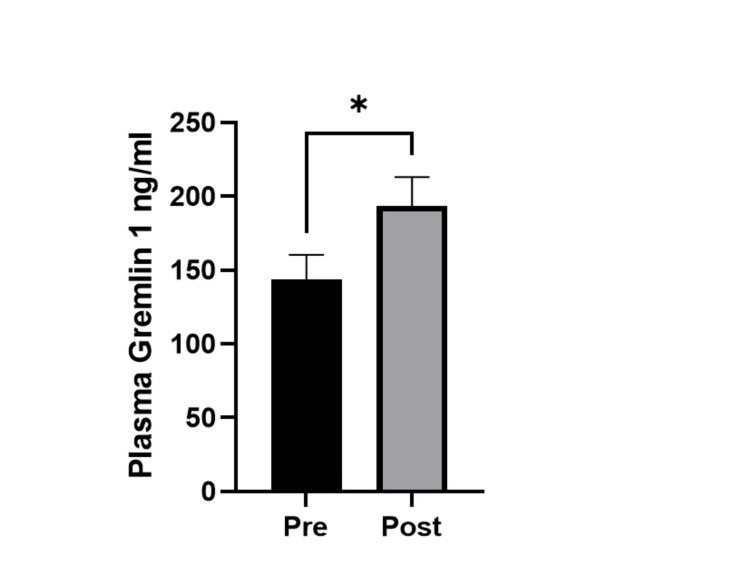
Plasma levels of Gremlin 1 before and after sleeve gastrectomy (n = 31) The error bars represent the standard error of the mean. *Indicates statistically significant differences (p < 0.05).

There was a significant increase (30%) in Gremlin 1 levels after sleeve gastrectomy (from 148.19±17.43 to 193.29±19.82 ng/mL, p < 0.05, Figure 1). Pearson correlation analysis revealed that Gremlin 1 positively correlated with insulin levels before surgery (r = 0.360, p < 0.05), whereas no correlation was observed after surgery (r = -0.009, Table 2).

**Table 2:** Pearson’s correlation of Gremlin 1 with clinical parameters Note: * Indicates significant correlation, p ≤ 0.05. GLUC, glucose; TGL, triglycerides; CHOL, cholesterol; HDL, high-density lipoprotein; LDL, low-density lipoprotein; HOMA-IR, homeostatic model assessment for insulin resistance; Corr, calcium-corrected; PTH, Parathyroid hormone.

Parameters		Before surgery (r)	After surgery (r)
Gremlin 1	GLUC	0.16	-0.08
	TGL	-0.02	-0.20
	CHOL	-0.03	0.03
	HDL	-0.04	-0.14
	LDL	-0.01	0.07
	Insulin	0.36^*^	-0.009
	HOMA-IR index	0.24	-0.06
	Albumin	0.13	-0.10
	Total calcium	0.18	-0.07
	Corrected calcium	0.14	0.24
	Phosphorus	0.18	0.27
	PTH	-0.14	-0.23
	Vitamin D	0.12	0.11

## Discussion

The aim of this study was to investigate the plasma level of Gremlin 1 in nondiabetic individuals with obesity undergoing sleeve gastrectomy and its relation to glycemic and lipid profiles. First, we aimed to investigate the changes in insulin resistance as reflected by HOMA-IR values following sleeve gastrectomy; a 56.55% decline in HOMA-IR values was noted among our participants, coupled with a 56.39% reduction in median circulating insulin levels, both of which support enhanced insulin sensitivity following bariatric surgery. A significant drop in BMI and triglyceride levels was also noted in our cohort; however, this was accompanied by an increase in HDL and cholesterol levels, all within the range of expected physiological levels (Table 2) [21-22]. We previously reported similar findings in which plasma cholesterol, HDL, and LDL levels were elevated within physiological values after sleeve gastrectomy, concomitant with increased levels of growth hormones, Insulin-like growth factor 1 (IGF-1), and IGF binding protein 2 (IGFBP-2) [23]. We partially attribute these lipid changes to improved growth hormone activity and increased fatty acid turnover in response to body weight reduction.

We measured the plasma levels of Gremlin 1 following bariatric surgery to prospectively correlate the changes with glycemic parameters. Gremlin 1 levels increased by 29.79% following sleeve gastrectomy. Our findings are not in agreement with those of a previous study in which a reduction in Gremlin 1 mRNA expression in adipose tissue (subcutaneous and visceral) was observed in obese individuals undergoing two-step bariatric surgery [20]. This demonstrates that improvement in insulin resistance can occur following bariatric surgery despite elevations in serum levels of Gremlin 1, an adipokine renounced for its antagonism of insulin action; this suggests that reduced levels of Gremlin 1 are not a prerequisite in improving insulin resistance following bariatric surgery. However, it is important to mention that we measured plasma protein levels instead of mRNA levels to investigate changes in Gremlin 1 levels, which could explain the discrepancy in the results. Elevated Gremlin 1 levels in obesity and T2D are reportedly associated with increased insulin resistance, as evidenced by an increased HOMA-IR index [20]. In the present study, a decrease in the HOMA-IR index was observed, suggesting increased insulin sensitivity despite the elevation in serum levels of Gremlin 1.

Circulating Gremlin 1 correlated positively with insulin levels before sleeve gastrectomy, which may indicate a role in insulin resistance. However, this correlation disappeared postoperatively, though this finding should ideally be replicated in a larger cohort. Gremlin 1 suppresses insulin action in primary adipocytes and attenuates basal- and insulin-stimulated glucose uptake, implying a role of Gremlin 1 in insulin resistance in obesity [20]. Similarly, we previously reported an association between plasma Gremlin 1 levels and body adiposity, and poor glycemic control [19]. However, after body weight reduction and changes in gut hormone levels after gastrectomy, there was no correlation between Gremlin 1 and insulin levels. This may indicate another role of Gremlin 1 in physiological homeostasis after bariatric surgery.

Obesity is associated with increased bone mineral density caused by increased mechanical loading, multiple hormonal factors including adipokines, and increased aromatization of androgens [24]. Moreover, several studies have reported the deleterious effects of bariatric surgery on bone metabolism, as indicated by increased bone turnover biomarkers, including C-terminal telopeptide of type 1 collagen and osteocalcin [24]. Reports have also documented decreased bone density and an increased risk of fracture after bariatric surgery [25]. These effects are presumably mediated by mechanical unloading and hormonal and nutritional factors [25]. In contrast, Gremlin 1 counters the effects of BMP on osteoblastic differentiation and function, and its overexpression causes osteopenia [26]. Furthermore, the deletion of grem1 improves BMP activity in the bone microenvironment and enhances bone formation [27]. In our cohort, Gremlin 1 levels increased after sleeve gastrectomy. Therefore, it is speculated that Gremlin 1 might also be involved in bone remodeling following bariatric surgery. 

Gremlin 1 loss in mice leads to mucosal abnormalities in the small and large intestines associated with malabsorption and bone marrow hypoplasia, suggesting a role of Gremlin 1 in maintaining normal bowel epithelial function in adulthood [28]. We speculated that increased plasma Gremlin 1 levels could be a compensatory mechanism for improving the well-being of the intestines and absorption after bariatric surgery. Moreover, Shahidi et al. reported that although the expression of Gremlin 1 increased in liver cells, its serum levels remained largely unchanged, indicating that the site of production of Gremlin 1 and its subsequent secretion in the bloodstream or a paracrine fashion predicts varying roles for Gremlin 1 in different tissues [29]. Furthermore, treatment of mouse adipocytes ex vivo with Gremlin 1 protein did not alter insulin signaling but inhibited BMP4 [29]. These findings add to the evidence that Gremlin 1 mRNA expression might not reflect its plasma levels and support our findings that a reduction in Gremlin 1 level might not be the causative factor for improving insulin sensitivity after sleeve gastrectomy but also adds an implication to future therapeutic uses as the benefit of elevated levels of Gremlin 1 after bariatric surgery remains unexplained, complicating the prospect of systemic Gremlin 1-based antagonism in the treatment of type 2 diabetes mellitus.

Interspecies differences were also observed in BMP4 antagonizing effects of Gremlin 1 in browning WAT in mice. No difference in body weight or browning of adipose tissue following transgenic expression of BMP4 was observed, which may be attributed to an increased level of Noggin, another BMP4 antagonist, thereby silencing the effects of BMP4 on WAT [30]. The aforementioned studies suggest that Gremlin 1 is part of an interaction network of BMP antagonists, with possible differences between humans and other mammalian species. Future studies should focus on these confounding factors.

There were some limitations to our study including the lack of control for patients who were lean or underwent unrelated laparoscopic surgery. This was a pre- and post-single arm study, a cohort design with the addition of more subjects will likely yield a more definitive inference of such findings. Additionally, despite showcasing an increase in serum levels of Gremlin 1, the reason for this increase and its physiological role remains unexplained though it can be speculated from the aforestated literature reports of a potential homeostatic role of Gremlin 1. Gremlin 1 is a relatively novel adipokine, and confoundedness cannot be excluded in those with comorbid health conditions or dietary inadherence. Moreover, this was a single-center study with 31 individuals, and the inclusion of more patients may allow for a correlative comparison between the metabolic variables with a statistically significant outcome, which was not possible in the present study, mainly because of the small sample size. Furthermore, a detailed analysis of Gremlin 1 in a larger cohort of individuals undergoing bariatric surgery by including females and taking into consideration bone and intestinal health, might provide a better understanding of the role of Gremlin 1 after bariatric surgeries.

## Conclusions

In conclusion, we report here that the improvement in insulin sensitivity after bariatric surgery can occur with elevated serum levels of Gremlin 1, despite its reported antagonistic effects; however, this would need to be reassessed with a larger cohort, which includes females and ideally control subjects. The aforementioned finding suggests that the improvement in insulin resistance in our cohort resulted from factors unrelated to serum levels of Gremlin 1, given its antagonistic effect on insulin function. It should, however, be borne in mind that interspecies differences may still exist with respect to their effects on adipose and possibly other tissues. Moreover, the possible function of Gremlin 1 in the physiological homeostasis of the alimentary tract adds another layer of complexity to ongoing studies and its potential therapeutic use in the treatment of type 2 diabetes mellitus. Though the potential benefit of Gremlin 1 antagonism cannot be excluded, the improvement in metabolic parameters of our cohort despite its elevation suggests that other biochemical alterations are likely more responsible for the beneficial effects of bariatric surgery.
